# An Atypical Case of Bladder Lipoma Presenting as Gross Hematuria: A Case Report

**DOI:** 10.7759/cureus.42471

**Published:** 2023-07-26

**Authors:** Aris Kaltsas, Athanasios Zachariou, Dung Mai Ba Tien, Atsushi Takenaka, Nikolaos Sofikitis

**Affiliations:** 1 Department of Urology, University of Ioannina, Ioannina, GRC; 2 Department of Andrology, Binh Dan Hospital, Ho Chi Minh City, VNM; 3 Division of Urology, Department of Surgery, School of Medicine, Faculty of Medicine, Tottori University, Yonago, JPN

**Keywords:** urology, mature adipocytes, transurethral resection, gross hematuria, bladder lipoma

## Abstract

While conventional lipomas represent the most commonly seen benign mesenchymal tumor in adults, their occurrence in the bladder wall is exceptionally rare. This report details a rare case of a bladder lipoma, a benign tumor primarily composed of mature adipocytes, presenting as gross hematuria in a 68-year-old male. Despite the patient's previous history of left nephrectomy, no significant pathological findings were initially observed. The bladder lipoma was detected via cystoscopy as a polypoid mass on the posterior bladder wall and confirmed through transurethral resection of the bladder tumor (TUR-BT). Histopathological analysis verified the mass as a bladder lipoma composed of mature adipocytes. Following a specific postoperative follow-up period, no recurrence of the tumor was observed, suggesting successful treatment. This case underscores the clinical significance of considering bladder lipoma in differential diagnoses of bladder tumors, especially in patients presenting with gross hematuria, given its exceptional rarity.

## Introduction

Lipomas, well-encapsulated benign tumors primarily composed of mature adipocytes, are the most frequently seen soft-tissue tumors in adults [1]. Their manifestation is most common in the superficial tissues of the proximal limbs and trunk, with a similar histopathological morphology observed across various types [2]. Although visceral lipomas are less common, they are not unheard of. An exceedingly rare subtype of these lipomas occurs within the urinary bladder [3]. Such bladder lipomas have been infrequently documented in the medical literature due to their nonspecific clinical presentation, often diagnosed via endoscopic biopsy, with further excision for clinical management typically not required [4]. This case report presents a unique instance of bladder lipoma in a patient presenting with gross hematuria, emphasizing the need for increased clinical awareness and consideration in differential diagnoses, which have also been described within the urinary bladder [5, 6].

## Case presentation

A 68-year-old male patient arrived at the Department of Urology, expressing concerns about gross hematuria. His medical history revealed a radical left nephrectomy conducted five years prior due to renal cell carcinoma, with no subsequent signs of recurrence. Notably, the patient was not receiving any form of anticoagulant or antiplatelet therapy.

To evaluate the cause of hematuria, initial laboratory tests were conducted. These included a complete blood count, liver function tests, kidney function tests, and a coagulation profile. All test results fell within the normal range, offering no immediate clues to the hematuria's source. An ultrasound examination was performed as part of the initial diagnostic process. However, the ultrasound also did not reveal any significant pathological findings.

**Figure 1:**
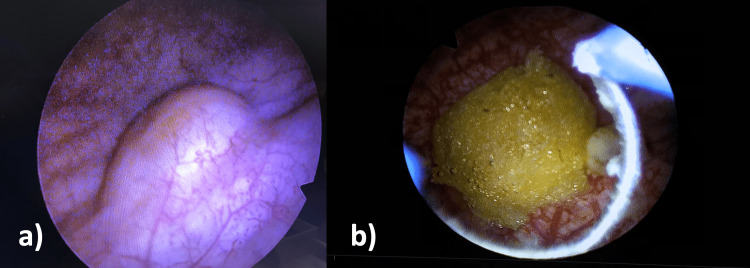
Cystoscopic views of the bladder lipoma (a) Pre-resection view showing the smooth, whitish, polypoid mass on the posterior bladder wall. (b) Mid-resection view revealing the homogeneous, yellowish tissue indicative of lipoma.

The next step in the diagnostic workup was a cystoscopy. This procedure uncovered a smooth, whitish, well-differentiated polypoid mass on the posterior bladder wall (Figure 1a).

Given this finding, a computerized tomography (CT) scan was performed to gain more insight into the nature of the mass and to exclude other potential etiologies of the hematuria. The CT scan showed mild thickening of the bladder wall and a fatty density, indicative of an intravesical extension of the mass, while, importantly, no other potential causes for the patient's hematuria were identified. Considering the patient's age, it was crucial to rule out potential gastrointestinal involvement as well. The patient underwent a colonoscopy following the CT scan, which produced no abnormal findings, thereby ruling out the gastrointestinal origins of the tumor.

Following the discovery of the mass during the cystoscopy and CT scan, the patient underwent transurethral resection of the bladder tumor (TUR-BT). During the operation, the tumor exhibited a homogeneous, yellowish color, lending further support to the suspicion of a benign etiology (Figure 1b and Video 1).

**Video 1:** Intraoperative video: transurethral resection of the bladder lipoma This intraoperative video demonstrates the process of transurethral resection of the bladder lipoma, illustrating the removal of the homogeneous, yellowish tumor from the posterior bladder wall.

**Figure 2:**
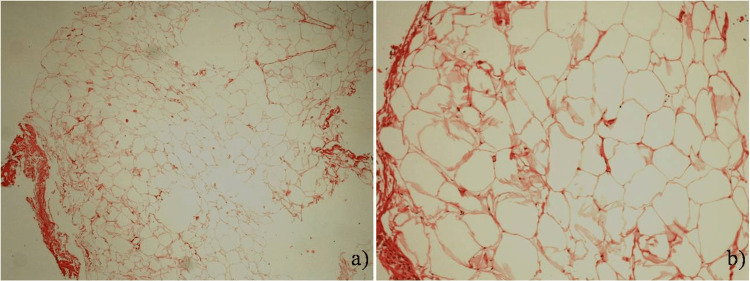
Microscopic evaluation of the bladder lipoma biopsy The biopsy samples were processed and stained with hematoxylin and eosin for cellular structure visualization. Panel A (x40 magnification) and Panel B (x100 magnification) display a comprehensive view of mature adipocytes, characteristic of lipoma.

Subsequent histopathological examination of the resected tissue revealed a well-encapsulated mass of mature adipocytes located within the lamina propria of the mucosa layer, a diagnosis consistent with a bladder lipoma (Figure 2).

There was no evidence of malignancy or bladder wall invasion, with the lesion encased by a thin rim of urothelial mucosa and no involvement of the muscularis propria. The absence of hyperchromasia or multinucleated stromal cells further ruled out a liposarcoma.

Postoperatively, the patient had an uneventful recovery and was hospitalized briefly before discharge. Regular follow-up examinations, including periodic cystoscopies and ultrasound evaluations, were scheduled. These follow-ups confirmed no recurrence of the bladder lipoma, marking the successful treatment of this rare condition.

## Discussion

A bladder lipoma is an extremely unusual tumor. Lipomas are a kind of benign tumor that develops from mature adipose tissue. Adult benign mesenchymal neoplasms are quite prevalent. Lipomas of the bladder are quite rare and make up a tiny fraction of all bladder tumors. In addition to the bladder, it's worth noting that lipomas have also been identified in the upper urinary tract, further indicating the rarity and wide-ranging occurrence of these tumors [7]. Lipomas of the bladder are uncommon and often cause no symptoms. They are often rather small, with lengths spanning only millimeters to centimeters at most [8].

The size and location of a bladder lipoma tumor may affect how the disease manifests clinically. In most cases, imaging or cystoscopy will uncover an asymptomatic bladder lipoma. Blood in the urine, urinary frequency, urgency, or dysuria (painful urination) are not always indications of a bladder lipoma, although they might be. These symptoms are not exclusive to bladder lipoma and may be present in a variety of bladder diseases [9,10].

Imaging examinations like ultrasonography, CT, or magnetic resonance imaging (MRI) are often utilized to diagnose and assess bladder lipomas. These imaging techniques may shed light on the tumor's extent, location, and phenotype. Typical imaging findings for bladder lipomas include hyperintense lesions on T1-weighted MRI images and well-defined, homogeneous, low-density masses on CT scans. However, in cases presenting with unusual characteristics, such as large size, necrosis, extension through the entire bladder wall, concerning features on imaging, or a personal or family history suggestive of a predisposition to sarcomas, a histopathological examination may be necessary. This examination provides a definitive diagnosis of bladder lipoma and helps exclude other mesenchymal neoplasms containing components of or resembling mature adipose tissue [11].

Lipomas of the bladder are often removed surgically. For lipomas that are small and close to the skin's surface, endoscopic excision is often the treatment of choice. Open surgical excision may be required if the lipoma is very large or deeply infiltrating. Importantly, lipomas of the bladder have an excellent prognosis given their benign nature, and it is worth highlighting that, contrary to the text's previous suggestion of low risk, they actually carry no risk of metastasis [12].

While the present case involves a bladder lipoma, it's worth noting that other rare benign tumors like bladder leiomyomas can also manifest with similar symptoms, warranting careful differential diagnosis to ensure appropriate treatment, as evidenced in a reported case of a 52-year-old man with a bladder trigone leiomyoma [6]. Well-differentiated liposarcoma, pelvic lipomatosis, and urachal fibrolipoma are all conditions that may be mistaken for bladder lipoma. Indicators of a well-differentiated liposarcoma include variation in adipocyte size, nuclear hyperchromasia, the presence of lipoblasts (even one might be enough to upgrade a diagnosis to atypical lipomatous neoplasm), and the presence of atypical multinucleated stromal cells [13]. Pelvic lipomatosis, characterized by an abnormal accumulation of mature adipose tissue in the perivesical and perirectal areas of the pelvic retroperitoneum, leads to external compression of the lower urinary tract and rectosigmoid colon [14]. Such conditions are often linked to germline alterations in retinoblastoma 1 (Rb1), leading to generalized lipomatosis [15]. Patients frequently report perineal or lower abdominal discomfort, increased urinary frequency, hematuria, and constipation, with fat deposition appearing patchy rather than nodular [12,16]. Infrequently, post-birth, the urachus may not involute fully into a fibrous cord, known as the median umbilical ligament, and calcified fibrolipomas can develop in this area [14]. No recurrences or sequelae have been reported from bladder lipomas, underscoring their benign nature and favorable prognosis [8].

In light of these findings, the key message we wish to convey is the necessity for urologists to maintain a high level of vigilance and a strong index of clinical suspicion for any bladder mass [5]. Even in the face of nonspecific symptoms and a negative lab or imaging workup, prompt diagnostic cystoscopy should not be delayed, enabling timely management [17]. This perspective also holds relevance when dealing with rare benign tumors like bladder lipomas.

## Conclusions

This case emphasizes the need to consider bladder lipomas in the differential diagnosis of bladder tumors, especially in patients presenting with gross hematuria. Early recognition and management can lead to excellent outcomes with no recurrence, as shown in this case. In conclusion, bladder lipoma is a very unusual benign bladder tumor. It has no noticeable symptoms and is generally discovered incidentally. Bladder lipomas may be evaluated using imaging investigations such as ultrasound, CT, or MRI, but a histological study is necessary for a definite diagnosis. The main therapy is surgical excision, and the prognosis is usually quite good. To better understand the clinical features, diagnosis, and therapy of bladder lipomas, further research and case reports are required.
